# Enhancing the flame retardancy of lyocell fabric finished with an efficient, halogen-free flame retardant

**DOI:** 10.1039/d1ra06573d

**Published:** 2021-10-28

**Authors:** Wei Tan, Yuanlin Ren, Mengyuan Xiao, Yingbin Guo, Yansong Liu, Jiayue Zhang, Xinke Zhou, Xiaohui Liu

**Affiliations:** School of Textile Science and Engineering, Tiangong University Tianjin 300387 China yuanlinr@163.com +86-22-8395-8287 +86-22-8395-5353; School of Materials Science and Engineering, Tiangong University Tianjin 300387 China xiaohuilau@163.com

## Abstract

A novel flame retardant (PNPG) containing phosphorus and nitrogen was synthesized through the reaction of neopentyl glycol, phosphoric acid and urea, and was then used for preparation of flame retardant lyocell fabric through a dip-dry-cure finishing process. The structure of the PNPG was confirmed by proton nuclear magnetic resonance spectroscopy (^1^H-NMR) and Fourier transform infrared spectroscopy (FT-IR). The flame retardancy and thermal stability of the treated fabric were evaluated by a cone calorimetry test and thermogravimetric analysis (TG), which showed that the char residue of the treated fabric at 800 °C was as high as 39.7% under a nitrogen atmosphere. At the same time, the peak heat release rate (PHRR) and total heat release (THR) were significantly reduced by 92.9% and 81.2%, respectively. Obviously, the presence of flame retardant can effectively improve the thermal stability and flame retardancy of lyocell fabrics. In addition, thermogravimetric analysis combined with Fourier transform infrared spectroscopy (TG-IR), scanning electron microscopy (SEM), and Raman spectroscopy indicated that the flame retardant mechanism was consistent with the condensed phase and gas phase mechanism. The limiting oxygen index (LOI) of the treated samples could reach 39.3%, moreover, even after 20 laundering cycles (LCs), the LOI values of the samples finished at 28.3% with 120 g L^−1^ flame retardant remaining, which confirmed the durability and high flame retardancy of the treated samples. In addition, the mechanical properties, whiteness, rigidity and flexibility of the fabrics treated with PNPG were insignificantly reduced within a more acceptable range than the original samples. In summary, the flame retardant described herein has excellent flame retardant properties and char-forming ability, and it is suitable for the preparation of flame retardant lyocell fibers.

## Abstract 2

A novel flame retardant (PNPG) containing phosphorus and nitrogen was synthesized based on neopentyl glycol, phosphoric acid and urea, and was then used for preparation of flame retardant lyocell fabric through a dip-dry-cure finishing process.

## Graphical abstract

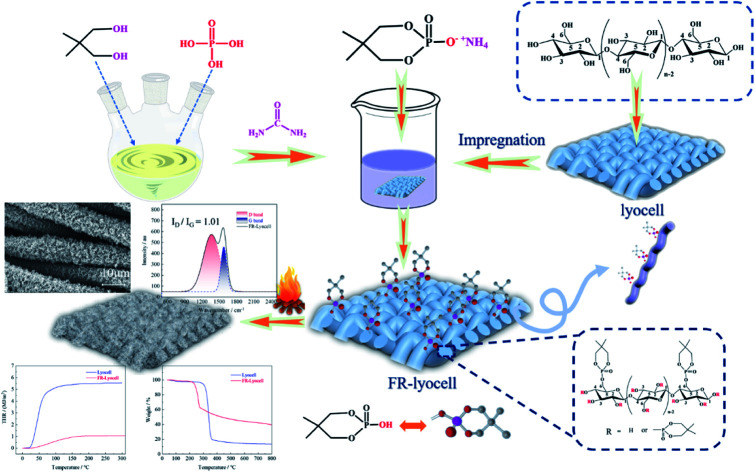

## 1. Introduction

Lyocell fiber, a kind of regenerated cellulose fiber, has the characteristics of comfort, softness, and easy dyeing. Specifically, the raw material for preparation of lyocell fiber, namely cellulose, is inexhaustible in nature.^1^ The production process of lyocell fiber is environmentally friendly, which is in line with the concept of green development in today's society.^2^ However, the limiting oxygen index (LOI) of lyocell fiber is about 18%,^3^ which means that the fiber is easy to burn and has a great potential fire hazard. Therefore, endowing lyocell fiber with flame retardancy is an effective solution to solve this issue.^4^

In terms of flame retardant modification, blend spinning, finishing processes and chemical grafting are the main methods for preparing flame retardant lyocell fiber.^5^ Blend spinning refers to mixing a suitable flame retardant with a spinning solution, then the resulting spinning solution is introduced into a coagulation bath through the spinneret, and then the flame retardant fiber is prepared by drawing.^6^ Delholm *et al.* mixed organically modified montmorillonite nanoparticles into a cotton cellulose/NMMO solution to significantly increase the char residue of the resulting regenerated cellulose during the combustion process. Specifically, when the clay additive was 15%, the residual char increased to 30%.^7^ Seddon *et al.* prepared flame retardant lyocell fibers with the LOI values up to 40% by doping *N*-hydroxymethyl-3-(dimethoxyphosphonyl) propionamide (Pyrovatex CP) during wet spinning.^8^ However, the blending technology requires good dispersion and compatibility of flame retardant in spinning solution. In addition, the amount of flame retardant also needs to be strictly controlled, because the mechanical properties of the prepared fiber will be deteriorated by excessive addition. Meanwhile, the spinnability of the spinning solution will also be affected.^9^ The finishing process is often utilized to improve the flame retardant performance of cellulose fibers, and the finishing system is usually composed of multiple components such as flame retardants, catalysts, crosslinking agents, and curing agents.^10^ Mengal *et al.* treated lyocell fibers with Provatex CP flame retardant and used citric acid as the crosslinking agent. The result showed that the lyocell fiber treated with the flame retardant with the concentration of 400 g L^−1^ had good flame retardancy even after 10 washing cycles, at the same time, the residual char reached 42%, indicating high char-forming efficiency.^11^ Nevertheless, the strong acidity of citric acid will cause great decrease in the fiber strength and affect its handle.^12^ Chemical grafting is the most commonly used flame retardant modification method for fiber, by virtue of the formation of covalent bonds between flame retardant molecules and fibers, the fibers with permanent flame retardant property is obtained.^13^ Tetrakis (hydrox-ymethyl) phosphoniumchloride (Proban)^14^ and Pyrovatex CP^15^ mentioned above are the representatives of commercial flame retardants for cellulosic fibers, which are already widely used in commercial production. The hydroxymethyl group in the flame retardant system forms the C–O–C bond with the hydroxyl groups of the cellulose molecular chains, which can stably link the two components. Unfortunately, in the process of preparation and using of flame retardant cellulose fibers or fabrics, carcinogenic formaldehyde will release, which seriously endangers human health.^16,17^ Therefore, it is imperative to design a formaldehyde-free, efficient and durable flame retardant.

In the last decades, halogen-based flame retardants had a large output and were widely used. However, some halogen-based flame retardants have a fatal flaw, that is, they release toxic and corrosive gases during the decomposition process. This is the reason why some halogen-based flame retardants are restricted or banned in many countries.^18^ In view of the shortcomings of the above-mentioned flame retardants, phosphorus-containing flame retardants have become a research hotspot in the field of flame retardancy due to their high efficiency and low toxicity.^19,20^ This is because phosphorus-based flame retardants can be pyrolyzed during the combustion process to produce phosphorous-containing derivatives as acid catalysts, thereby promoting the dehydration of the cellulose matrix into char, which as a result protects the cellulose from heat transfer and the release of combustible volatiles, and enhances the self-extinguishing property of the fiber matrix.

Based on the chemical reaction of neopentyl glycol (NPG) and phosphorus oxychloride, a highly effective flame retardant was synthesized to improve the flame retardancy of polymers.^21,22^ However, phosphorus oxychloride is not only expensive, but also potentially dangerous due to the toxic, irritating, and corrosive gases emitted.^23^ To solve the issue, can low-cost, non-toxic phosphoric acid be used as a substitute for phosphorus oxychloride in the synthesis of flame retardant?

In this work, an efficient and environmentally friendly halogen-free phosphorus-based flame retardant was designed and synthesized by the reaction of NPG and phosphoric acid, which can be covalently grafted to lyocell fiber or its fabric by P–O–C to obtain durable flame retardancy. The structure and surface morphology of the modified fiber were researched in detail, moreover, the flame retardant and combustion behaviour were also investigated by a variety of analytical techniques. The flame retardant mechanism was explored.

## 2. Experimental section

### 2.1 Materials

Neopentyl glycol (NPG) was supplied by Aladdin Chemistry Industry Co. Ltd (Shanghai, China). Absolute ethanol, urea, and phosphoric acid (H_3_PO_4_, 85 wt%) were obtained from Tianjin Guangfu Fine Chemical Research Institute (Tianjin, China). Lyocell fabrics were got from Tiangong University (Tianjin, China). The reagents were of analytical grade and were used directly as received.

### 2.2 Preparation of neopentyl glycol-based flame retardant

**Fig. 1:**
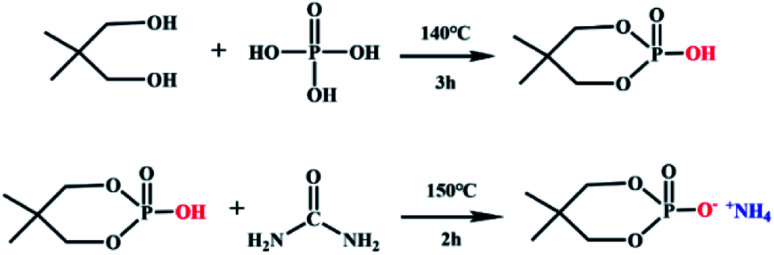
Synthesis route of the PNPG.

NPG (0.15 mol, 15.6 g) and phosphoric acid (0.15 mol, 17.3 g) were introduced into a three-necked flask equipped with a condenser and reacted at 140 °C for 3 h. Then, urea (0.2 mol, 12.0 g) was poured to the reaction system, at the same time, the temperature was raised to 150 °C for 1 h to obtain a pale-yellow liquid. It was washed 3 times with absolute ethanol, and white crystals were obtained by suction filtration. The synthesis schematic diagram of neopentyl glycol-based flame retardant was shown in Fig. 1.

### 2.3 Fabricating of flame retardant lyocell fabrics (FR-lyocell)

**Fig. 2:**
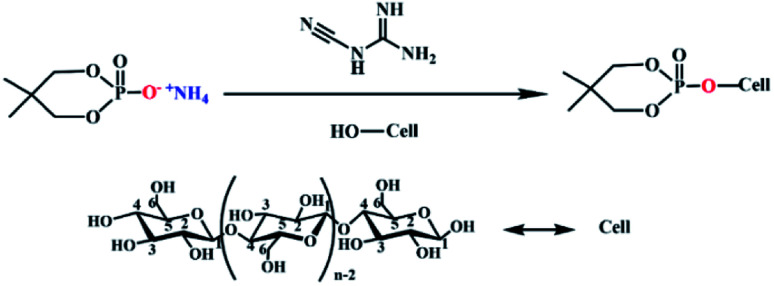
Preparation of FR-lyocell sample.

The prepared flame retardant was dissolved in deionized water at a concentration of 120 g L^−1^, and dicyandiamide accounted for 7% of the mass of the flame retardant was used as a catalyst was added to promote the formation of covalent bonds. The lyocell fabric was immersed in finishing liquid and stirred in a water bath at 70 °C at a bath ratio of 1 : 35 for 3 h to reach wet pickup of about 60%. The fabric was placed in a constant temperature oven at 165 °C for 7 min. The schematic diagram of grafting was shown in Fig. 2.

The grafting rate (GR) of FR-lyocell was calculated by the following formula:GR (%) = (*W*_2_ − *W*_1_)/*W*_1_ × 100%where *W*_1_ and *W*_2_ were the weights of the blank and modified fibers, respectively.

### 2.4 Characterization

The chemical structure of PNPG was verified by proton nuclear magnetic resonance (^1^H-NMR, Bruker Corporation, Germany) spectroscopy. Deuterium oxide (D_2_O) and tetramethylsilane (TMS) were used as the solvent, and the internal standard.

FT-IR spectra of PNPG, control and modified fabric were obtained by a Spectrum GX spectrometer (Thermo Fisher Scientific Inc., China) using a KBr disk in the range of 400–4000 cm^−1^.

X-ray photoelectron spectroscopy (XPS) was performed to examine the element compositions and content of PNPG, pristine sample and treated sample *via* AXIS-Ultra DLD XPS spectrometer (K-alpha, USA).

The thermal performance of the control sample and the treated sample was measured by using a thermogravimetric analyzer (PerkinElmer, USA) from 40 °C to 800 °C at 10 °C min^−1^ in air and nitrogen atmosphere, respectively.

TG-IR spectra of the virgin and treated lyocell samples were carried out on STA 6000 thermogravimetry analyzer (PerkinElmer, USA) connected to FT-IR spectrophotometer. About 5.0 mg of samples were placed in an alumina crucible and heated from 40 °C to 900 °C with the heating rate of 20 °C min^−1^ and the nitrogen flow rate of 30 mL min^−1^.

The surface morphology and element distribution of the original sample and the processed sample were characterized by a scanning electron microscope (SEM, Zeuss, Japan) equipped with an energy dispersive spectrometer (EDS). The samples were deposited with platinum by a sputter coater before observation.

The combustion performance of the control and treated fabric (100 mm × 100 mm × 2 mm) in the horizontal configuration was evaluated by cone calorimetry (FTT 0007, UK) with a radiant heat flux of 35 kW m^−2^. In light of the GB/T5455-1997 standard, the burning performance of the control and FR-lyocell fabrics was obtained.

Raman (DXR2, Thermo Scientific, America) spectroscopy was used to evaluate the residual char of the treated sample to determine the degree of graphitization. The measurement interval was 500–2500 cm^−1^, and the resolution was 1 cm^−1^.

Durability tests of the treated samples were performed according to AATCC-61-2003 standard. The detergent concentration was 3.7 g L^−1^, and the single laundering time was 45 minutes.

The LOI values of samples were tested by using JF-5 automatic oxygen index analyzer (Nanjing Jionglei Instrument & Equipment Co., Ltd, China). In accordance with GB/T5454-1997 standard, the samples with dimensions of 150 mm × 58 mm were performed under different LCs.

The tensile strength and elongation at break of the samples were tested on YG065H250/pc electronic fabric strength machine (Laizhou Electronic Instruments Co., Ltd China) according to GB/T3923.1-1997 standard.

The whiteness of the original and treated samples was investigated *via* a WSD-3U fluorescence whiteness meter (Beijing instrument KangGuang optical instrument Co., Ltd China) with the dimensions of 200 mm × 50 mm based on the standard GB/T 17644-2008.

The stiffness and flexibility of the samples were carried out on the YG (B) 022D type automatic fabric stiffness tester (Shaoxing Yuanmore Electrome-chanical Equipment Co., Ltd China), according to ASTM D 1388-96 (2002).

## 3. Results and discussion

### 3.1 Characterization of PNPG

**Fig. 3:**
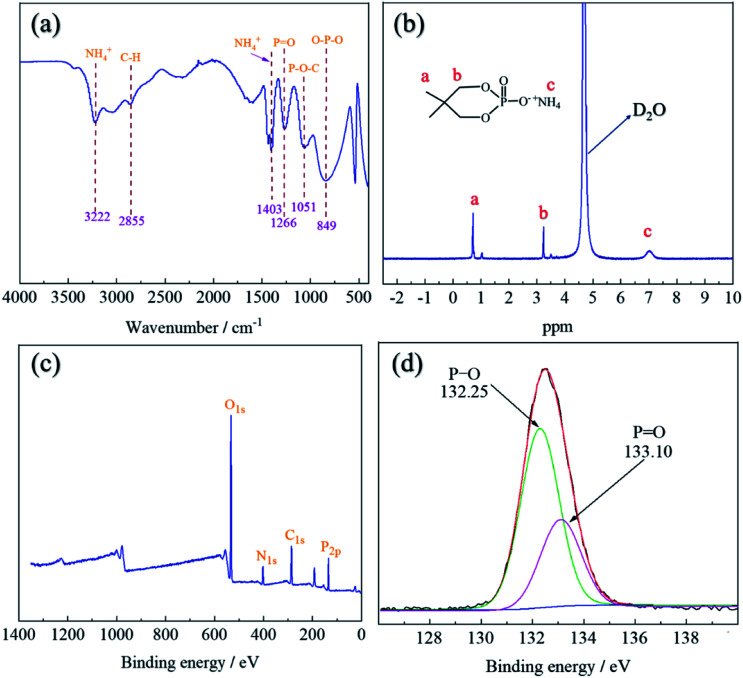
FT-IR spectrum (a), ^1^H-NMR spectrum (b), total XPS spectra (c) and high-resolution P_2p_ spectra (d) of PNPG.

The structure of PNPG was characterized by FT-IR spectra, as shown in Fig. 3(a). The narrow peaks at 3222 cm^−1^ and 1403 cm^−1^ were the stretching vibration and scissoring vibration absorption bands of NH_4_^+^.^24^ The characteristic peak at 2855 cm^−1^ was due to the stretching vibration of C–H.^25^ The peak at 1266 cm^−1^ was attributed to P=O groups.^26^ Furthermore, the peaks of P–O–C and O–P–O were at 1051 cm^−1^ and 849 cm^−1^.^24^ The FT-IR results indicated the existence of the characteristic absorption peak of PNPG.

The chemical structure of PNPG was further confirmed by ^1^H-NMR spectroscopy, as shown in Fig. 3(b). The peak that appeared at 0.73 ppm belonged to the hydrogen shift of –CH_3_ [(CH_3_)_2_–C, Ha], and 3.25 ppm was attributed to the hydrogen of –CH_2_ (P–O–CH_2_, Hb). The peak at 4.72 ppm was ascribed to the hydrogen of the deuterated solvent (D_2_O). In addition, the chemical shift of the proton in NH_4_^+^(POO–NH_4_^+^, Hc) was detected at 7.05 ppm. The appearance of these peaks coincided with the characteristic groups contained in the PNPG structure.

In order to further explore the surface chemical composition of the PNPG, XPS was carried out. As illustrated in Fig. 3(c), the results showed that the main elements of flame retardant include C (C_1s_, 286.25 eV), O (O_1s_, 532.75 eV), N (N_1s_, 399.62 eV), and P (P_2p_, 133.21 eV), the positions of the characteristic peaks of these elements were in line with previous research.^27–29^ For the P_2p_ spectra (Fig. 3(d)), the peak with the binding energy of 133.10 eV represented the P=O functional group, and the peak with the binding energy of 132.25 eV was attributed to P–O,^30^ which were consistent with the results of FT-IR analysis. Therefore, the above results indicated that the PNPG flame retardant has been synthesized successfully (Table 1).

**Table 1:** XPS elements analysis of PNPG

Element	N_1s_	P_2p_	C_1s_	O_1s_
Atomic%	7.31	13.83	29.31	49.55

### 3.2 FT-IR analysis

**Fig. 4:**
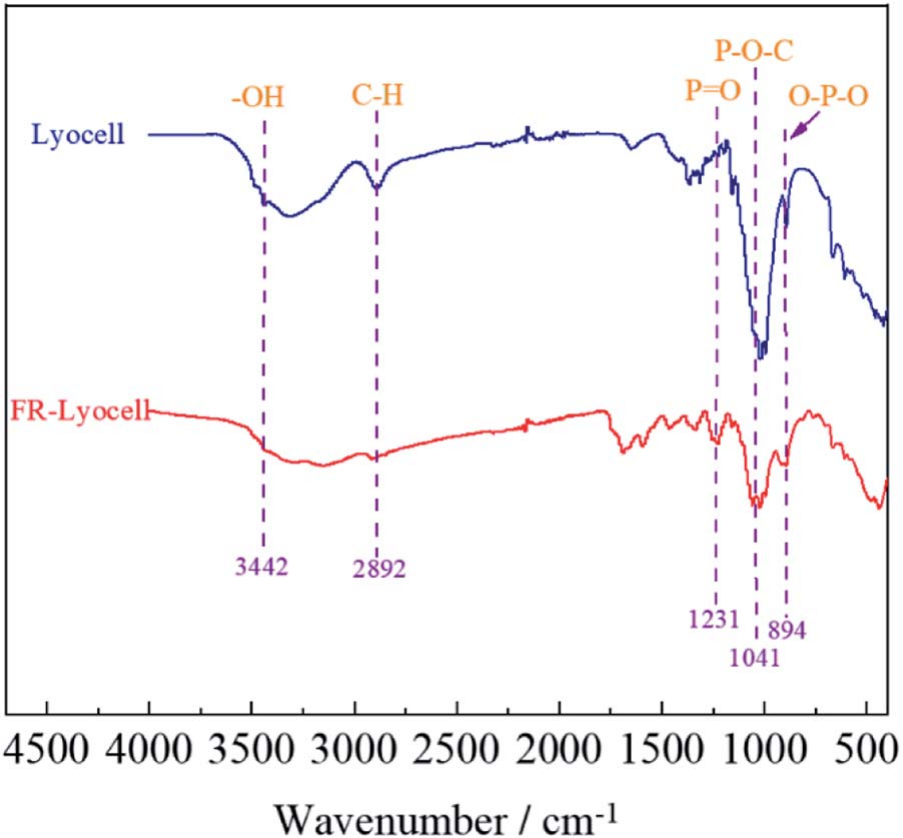
FT-IR spectra of the lyocell and FR-lyocell fabrics.

The FT-IR spectra of lyocell and FR-lyocell samples were displayed in Fig. 4. For the two samples, the peak located at 3442 cm^−1^ was due to the stretching vibrations of the typical functional group of –OH.^31^ The characteristic peak of 2892 cm^−1^ was attributed to the vibration of C–H.^32^ For FR-lyocell, the characteristic peak at 1231 cm^−1^ corresponded to the P=O functional group.^33,34^ The peaks of the P–O–C and O–P–O groups were appeared at 1041 cm^−1^ and 894 cm^−1^.^35^ In other words, these newly-appeared absorption peaks indicated that the flame retardant molecules were successfully bonded to the lyocell fiber through the P–O–C covalent bond.

### 3.3 XPS analysis

**Fig. 5:**
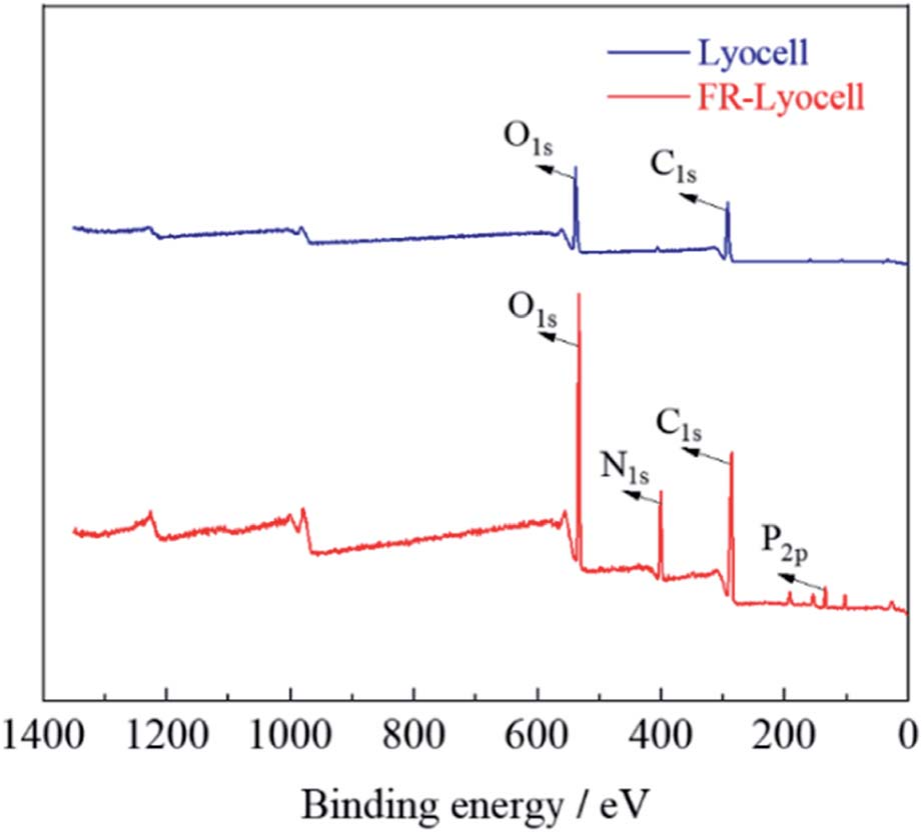
XPS spectra of lyocell and FR-lyocell.

XPS was performed to analyse the elemental composition and contents of the control and treated samples. As displayed in Fig. 5 and Table 2, the peaks at 286 eV and 533 eV corresponded to the C and O elements of the two fabrics,^36^ respectively. For the treated lyocell sample, the new characteristic bands appeared at 400 eV and 134 eV were ascribed to N_1s_ and P_2p_,^37^ resulting from the introduction of flame retardant molecules. In general, the amount of phosphorus contents is the main factor to determine the flame retardant efficiency,^38^ and the phosphorus content of about 1.5–4% can impart fiber excellent self-extinguishing properties.^39^ The phosphorus content of the samples prepared in this work was as high as 3.17%, which endowed the samples with excellent flame retardancy.

**Table 2:** Elemental contents of lyocell and FR-lyocell

Sample	C (at%)	O (at%)	N (at%)	P (at%)
Lyocell	64.49	30.58	—	—
FR-lyocell	52.22	28.85	12.77	3.17

### 3.4 Thermal stability

**Fig. 6:**
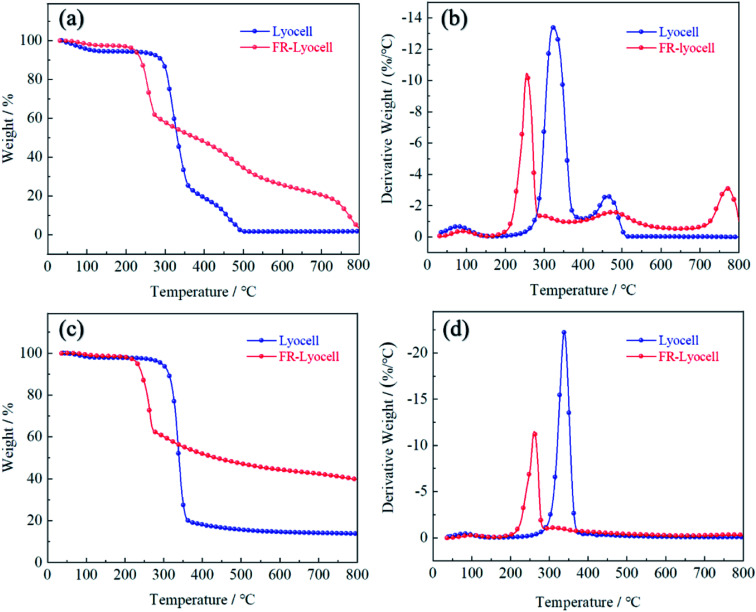
TG and DTG curves of the lyocell and FR-lyocell in air (a and b) and in nitrogen (c and d).

Thermogravimetric analysis (TG) was performed on the lyocell fabric and the treated lyocell fabric to evaluate their thermal properties, as shown in Fig. 6. The pyrolysis of lyocell fabric could be divided into three stages: initial decomposition, main decomposition and char decomposition. The initial decomposition occurred below 280 °C, attributing to the vaporization of free water attached to the surface of the fabric. The main decomposition step of the control sample occurred at 280–380 °C, either under air or nitrogen atmosphere, involving the degradation of cellulose molecular chains and the generation of fatty coke.^6^ The weight loss was 71.18% and 77.65% under air and nitrogen atmospheres, and the maximum weight loss rates were reached at 323 °C and 338 °C, respectively. The residual char of the fabric was further decomposed above 380 °C in which some of the aliphatic coal coke was converted to aromatic coal coke.^37,40^ Finally, the residual char of the control samples under air was 1.4% and 13.6% at 800 °C, respectively.

Compared with the original sample, the main weight loss stage of the modified lyocell sample was at 180–290 °C, with 38.03% and 37.18% mass losses under air and nitrogen atmosphere, respectively, which might be due to the lower stability of the P–O–C bond than the C–C bond,^41^ leading to the early degradation of phosphorus-containing compounds to form phosphoric and polyphosphoric acids, as a result, they could accelerate the dehydration and promote the formation of coke.^42^ Therefore, the phosphorus-rich residual char could act as an effective physical barrier to isolate the substrate from heat and oxygen and increase the amount of residual char.^43,44^ As shown in Table 3, the treated samples had higher residual char and lower thermal degradation temperature than that of the original sample, whether under oxygen or nitrogen conditions. These results demonstrated that PNPG could effectively accelerate the charring of lyocell fabrics, thus effectively ensuring the thermal stability of the substrates.

**Table 3:** TG data of the samples in air and nitrogen atmosphere

Test atmosphere	Samples	*T*_5%_ (°C)	*T*_50%_ (°C)	*T*_max%_ (°C)	Residue at 800 °C (wt%)
Air	Lyocell	111	330	323	1.4%
	FR-lyocell	222	376	255	3.8%
N_2_	Lyocell	294	339	338	13.6%
	FR-lyocell	232	433	260	39.7%

### 3.5 LOI and durability analysis

The LOI test was performed to evaluate the flame retardancy and durability of the treated samples, and the correlation between LOI values and durability of the fabrics treated with different flame retardant concentrations was summarized in Table 4. The LOI value of the original sample was only 18.2%, which belonged to the category of flammable materials. However, the LOI values of the samples modified with 120 g L^−1^, 150 g L^−1^ and 180 g L^−1^ flame retardant were as high as 36.4%, 37.8% and 39.3%, respectively. The results showed that the LOI values of the treated fabrics tended to increase with the increase of flame retardant concentration. However, with the increase of laundering cycles (LCs), the LOI values of the treated samples decreased to 28.3%, 30.7%, and 32.2%, respectively, which might be due to the loss of the flame retardant molecules that penetrated into the amorphous zone of the fibers under the mechanical friction. Fortunately, the treated samples still met the flame retardant category in terms of LOI value, even after 20 LCs. Therefore, the samples treated with PNPG had excellent flame retardant durability.

**Table 4:** The LOI values of the control and treated samples with different FR concentrations before and after different LCs

Sample	LOI (%)				
	0 LCs	5 LCs	10 LCs	15 LCs	20 LCs
Control lyocell	18.2	—	—	—	—
FR-lyocell-120 g L^−1^	36.4	34.8	32.7	30.4	28.3
FR-lyocell-150 g L^−1^	37.8	36.0	34.1	32.6	30.7
FR-lyocell-180 g L^−1^	39.3	37.6	35.5	33.8	32.2

### 3.6 Physical property

**Fig. 7:**
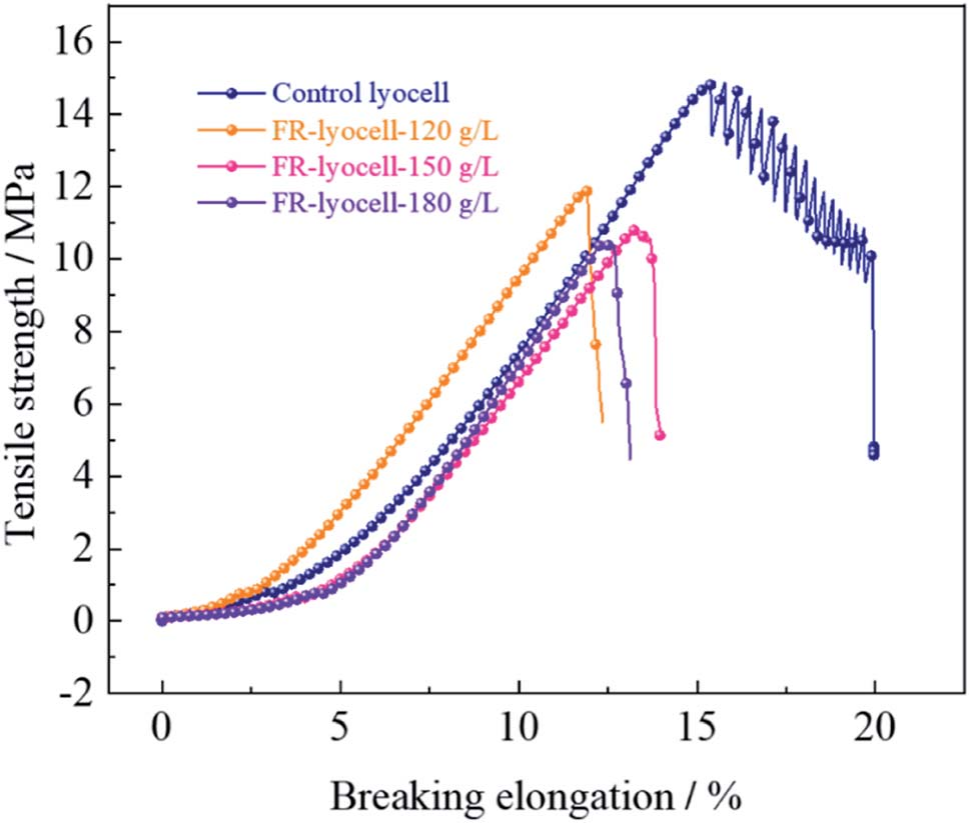
Mechanical properties of the lyocell and FR-lyocell fabrics treated with different FR concentrations.

The physical properties, such as mechanical properties and whiteness of the fabrics treated with PNPG have an important influence on the practical application. The physical properties of the original and modified samples were evaluated and the corresponding results were summarized in Fig. 7 and Table 5. Compared to the control sample, the tensile strength of the fabrics treated with 120 g L^−1^, 150 g L^−1^ and 180 g L^−1^ flame retardant decreased from 14.86 MPa to 11.96 MPa, 10.83 MPa and 10.41 MPa, while the breaking elongation decreased from 15.72% to 11.86%, 13.26% and 12.36%, respectively. Clearly, with the increase of flame retardant concentration, the tensile strength and breaking elongation of the treated samples showed a slight decrease trend. This might be due to the fact that the covalent bond formed between the flame retardant molecules and the cellulose molecular chains broke the hydrogen bonding network of the cellulose molecular chains so as to weaken the strong force between the cellulose molecules. In addition, the cellulose molecular chains could degrade under weak acidic environment of the flame retardant finishing solution, resulting in the decrease of mechanical properties.

**Table 5:** Mechanical properties of the control lyocell and FR-treated lyocell fabric

Sample	Tensile strength (MPa)	Breaking elongation (%)	Bending rigidity (g m^−2^)	Whiteness index (%)
Control lyocell	14.86 (±0.42)	15.72 (±0.22)	2.127	86.62
FR-lyocell-120 g L^−1^	11.96 (±0.27)	11.86 (±0.14)	2.183	76.80
FR-lyocell-150 g L^−1^	10.83 (±0.31)	13.26 (±0.12)	2.254	74.55
FR-lyocell-180 g L^−1^	10.41 (±0.28)	12.36 (±0.18)	2.281	71.96

Bending rigidity refers to the ability of the material to resist shape changes in the direction of bending, which is commonly used to describe the stiffness and flexibility of the fabric. With the increase of flame retardant concentration, the bending rigidity of the treated samples showed slight variations compared with the original samples, which indicated that the stiffness and flexibility of the treated samples did not change significantly and the hand feeling remained well. Besides, the whiteness of the modified samples decreased from 86.62% to 71.96%, suggesting the process of flame retardant treatment had little effect on the whiteness.

In conclusion, the treated samples had good physical properties and whiteness, and potential application in the textile field.

### 3.7 Cone calorimetry test

**Fig. 8:**
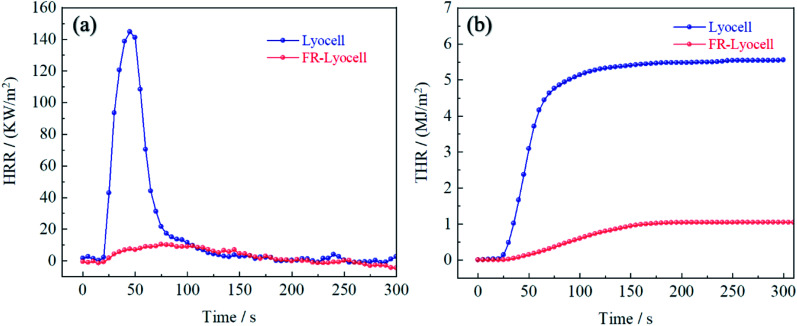
The HRR (a) and THR (b) curves of lyocell and FR-lyocell fabrics.

Cone calorimetry was carried out to accurately assess the combustion properties of the control and FR-lyocell fabric. The curves and the main data were exhibited in Fig. 8 and Table 6. The heat release rate (HRR) and total heat release (THR) values of the treated fabric were 10.3 kW m^−2^ and 1.0 MJ m^−2^, respectively, which were significantly lower than that of the original sample. This meant that less heat was fed back to the surface of the material during the combustion process, which resulted in a decrease in the pyrolysis rate of the matrix, thereby delaying the spread of flames. In light of the time to peak of heat release rate (TTPHRR) of the two samples, the original fabric was 45 s, while the modified fabric extended to 75 s. This could explain why the presence of PNPG greatly delayed the combustion process. In other words, PNPG could prevent the fabric from burning rapidly thereby gaining more response time for fire rescue. The final residual char of the original fabric was 9.8%. As expected, the residual char of the treated fabric was as high as 20%. The high residual char was consistent with the flame retardant mechanism of the condensed phase.^45^ The total smoke production (TSP) of the modified fabric was higher than that of the blank sample, which might be due to the fact that the dense graphitized char layer isolated heat and oxygen, resulting in insufficient combustion.

**Table 6:** Cone calorimeter data of the lyocell and FR-lyocell fabrics

Sample	TTI (s)	PHRR (kW m^−2^)	TTPHRR (s)	THR (MJ m^−2^)	TSP (m^2^)	FIGRA (kW m^−2^ s^−1^)	Residue (%)
Lyocell	18	144.7	45	5.5	0.0	3.21	9.8%
FR-lyocell	Unignite	10.3	75	1.0	0.5	0.14	20.0%

The fire growth rate index (FIGRA) is the key parameter for appraising the flame retardant properties of materials in a real fire, which is defined as the ratio of PHRR and TTPHRR.^46^ The FIGRA of the modified fabric dropped sharply from 3.21 to 0.14 kW m^−2^ s^−1^, which meant that the fire safety performance of the FR-lyocell was higher.

### 3.8 TG-IR analysis

**Fig. 9:**
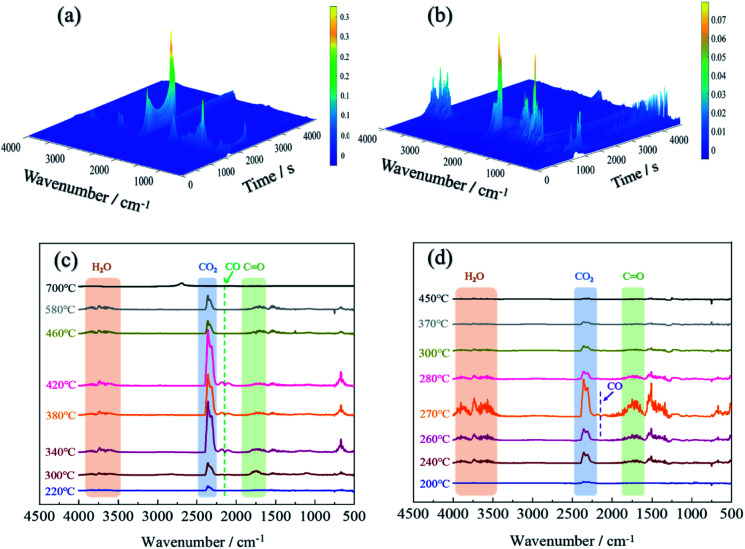
3D TG-IR spectra of the pyrolysis volatiles for lyocell fibers (a) and FR-lyocell (b); FTIR spectra of the pyrolysis products for lyocell fibers (c) and FR-lyocell (d) at different temperatures.

TG-IR analysis can better understand the mechanism of thermal degradation and identify the composition and structure of volatiles.^47^ The TG-IR spectrum of the original fabric was shown in Fig. 9(a) and (c). The peaks at 3500–4000 cm^−1^ were attributed to the stretching vibration of –OH, which was derived from water vapor produced during thermal degradation.^48^ The sharp characteristic band at 2360 cm^−1^ corresponded to CO_2_,^49^ which initially appeared at 220 °C, and the peak intensity reached its maximum at 340 °C and finally disappeared at 580 °C. When the temperature was between 340–420 °C, a weak absorption peak appeared at 2144 cm^−1^, which implied the formation of carbon monoxide.^50^ The strong peak near 1745 cm^−1^ was assigned to the C=O of carbonyl compounds, which was considered to the pyrolysis product of levoglucosan.^51^

For the FR-lyocell, the results were shown in Fig. 9(b) and (d). When the temperature was between 240-370 °C, the characteristic peaks at 3500–4000 cm^−1^ and 2360 cm^−1^ were the stretching vibration peak of –OH and the contraction vibration peak of carbon dioxide.^52^ The characteristic peaks at 1745 cm^−1^ and 2144 cm^−1^ were attributed to C=O and carbon monoxide,^21,50^ and their absorption intensity was significantly lower than that of the original sample. Therefore, it can be inferred that the released carbon monoxide and carbonyl-containing volatile products of the treated fabric were significantly reduced. This might be due to the fact that the produced dense char layer inhibited the release of volatile products.^53^ Compared with the original sample, the characteristic band of the modified sample nearly disappeared at 370 °C, which was much lower than that of the original sample. This proved that the flame retardant molecules inhibited the pyrolysis process and improved the pyrolysis process to move to lower temperature. At the same time, the maximum absorption peak strength of the gaseous pyrolysis products was significantly reduced, which contributed to the enhancement of the flame retardancy and thermal stability of the fabric.

### 3.9 SEM and EDS analysis

**Fig. 10:**
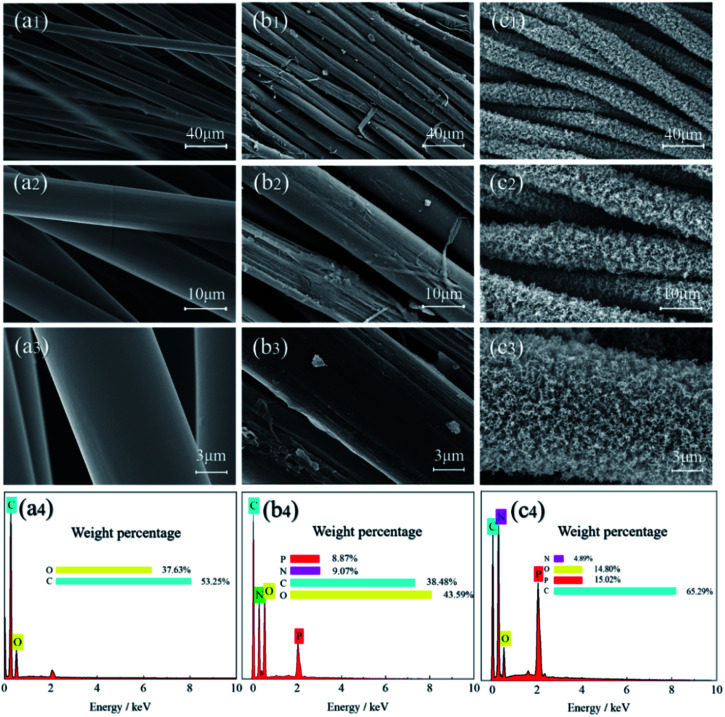
SEM images and EDS data of the control fabric [(a1)–(a4)], FR-lyocell fabric [(b1)–(b4)], and the char residue of FR-lyocell fabric [(c1)–(c4)].

The surface morphology of the original and treated samples was observed by scanning electron microscope (SEM), as shown in Fig. 10. The surface of the blank sample (Fig. 10a1–a3) was clean and smooth without attachments. In contrast, the surface of FR-lyocell (Fig. 10b1–b3) was slightly coarse and accompanied by attachments. After the treated fabric was fully burned, the char residue became much coarse and remained intact (Fig. 10c1–c3). The dense and continuous char isolated the exchange of heat and oxygen so as to improve the thermal stability and flame retardancy of the substrate.^54^

Energy dispersive spectrometer (EDS) was used to measure the elemental composition and content of the samples, and the corresponding data were listed in Fig. 10(a4–c4). Only two elements, *i.e.*, carbon and oxygen were detected in the original sample. However, the presence of nitrogen and phosphorus in the treated fabrics was attributed to the introduction of flame retardant molecules. Compared with the treated fabric, the high content of carbon and phosphorus elements in the residual char suggested that the phosphorus-containing derivatives promoted the dehydration of the fabric matrix into char, while the phosphorus elements played a role in the condensation phase and remained in the stable char layer.^55^ The thermal insulation effect of the phosphorus-rich char layer helped to ameliorate the flame retardant property of the modified fabric.

### 3.10 Raman spectroscopy analysis

**Fig. 11:**
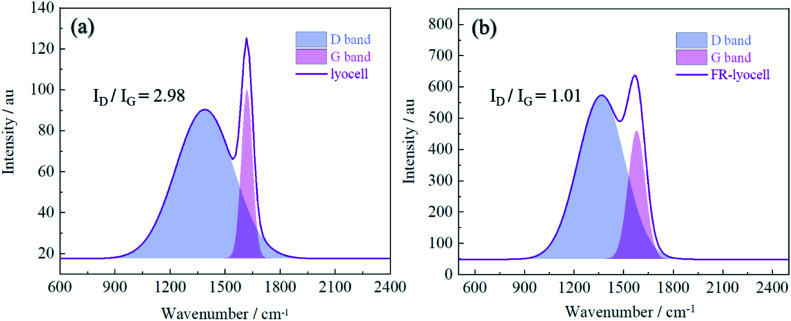
Raman spectra of the char residue of lyocell fibers (a) and FR-lyocell (b).

Raman spectrometer was utilized to characterize the residue char of the modified lyocell sample after combustion in order to further analyse the flame retardant mechanism. As displayed in Fig. 11, the Raman spectrum consists of two overlapping peaks, corresponding to the amorphous carbon at 1360 cm^−1^ (D band)^56^ and the graphitic carbon at 1587 cm^−1^ (G band),^25^ the ratio of the intensity of D band and G band (*I*_D_/*I*_G_) can reflect the degree of graphitization of the char layer.^57^ In general, the lower *I*_D_/*I*_G_ value implies the higher degree of graphitization, and the char layer is denser, the thermal stability is higher, indicating better barrier effect. The *I*_D_/*I*_G_ value of the original sample was 2.98, however, the *I*_D_/*I*_G_ value of the treated sample decreased to 1.01. Therefore, it could be concluded that PNPG could contribute to the transformation of amorphous carbon to graphitized carbon, as reflected by the efficient shielding effect and thermal oxidation stabilization.

### 3.11 Flame retardant mechanism analysis

**Fig. 12:**
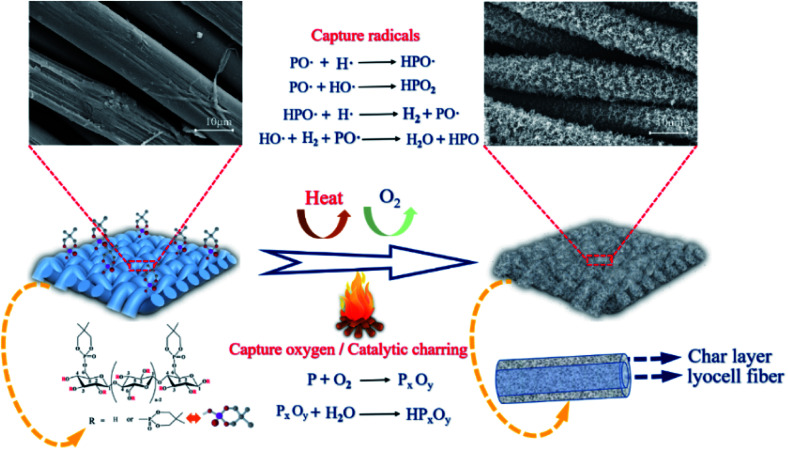
The illustration of the flame retardant mechanism.

Based on the above-mentioned experimental results, the possible flame-retardant mechanism of FR-lyocell fabrics was proposed in Fig. 12. FR-lyocell produces phosphorus-rich compounds at high temperatures, including phosphoric acid and polyphosphoric acid.^58^ The phosphorus-rich composition generated from the chemical reactions such as high temperature esterification and cross-linking mainly acted as a physical barrier in the condensed phase,^54^ which isolated the exchange of oxygen and heat, and inhibited the further thermal oxidation of the matrix in the combustion zone. This was consistent with the condensed phase flame-retardant mechanism. In addition, the treated samples might also form some of the reactive radicals (PO˙ and HPO˙) during the combustion process, which could capture the H˙ and HO˙ free radicals generated from the fiber pyrolysis to terminate the free radical chain growth reaction in the combustion process,^59^ as a result, the treated samples not only reduced the heat release and the thermal feedback to the underlying fiber matrix but also inhibited the production of combustible compounds, which was consistent with the gas-phase flame retardant mechanism.^60^ In a word, PNPG endowed lyocell fabrics with high-efficiency flame retardancy due to the synergistic condensed phase and gas phase flame-retardant role.

## 4. Conclusions

A high-efficiency, halogen-free, and formaldehyde-free flame retardant was successfully synthesized and applied to the flame-retardant modification of lyocell fabrics. ^1^H-NMR and FT-IR analysis proved that the flame retardant was successfully prepared. FT-IR and XPS analysis indicated that the flame retardant molecules were grafted onto the lyocell fabric through the P–O–C covalent bond. At the same time, the high phosphorus content provided a guarantee for the flame retardancy of lyocell fibers. TG results revealed that the thermal stability and char forming ability of the treated fabrics were improved whether in nitrogen or air conditions. The LOI values of the treated samples still remained above 28.3% even after 20 LCs, indicating good durable flame retardancy. The results of cone calorimetry demonstrated that the PHRR and THR values of the treated fabric were significantly reduced, indicating that the flame retardant inhibited the heat release of the fabric during the combustion process. The TG-FTIR, SEM, and Raman spectroscopy suggested that the flame retardant acted synergistically in the condensed phase and the gas phase. In addition, the physical properties of the modified samples, such as mechanical properties, stiffness and flexibility, and whiteness, were slightly reduced within an acceptable range. Based on the above experimental results, it can be concluded that this novel flame retardant provides a new strategy for the flame retardancy of lyocell fabrics.

## Author contributions

Wei Tan and Yuanlin Ren came up with the idea and performed specific experiments. Mengyuan Xiao and Yingbin Guo did preliminary investigation and mechanism analysis. Yansong Liu, Jiayue Zhang and Xinke Zhou analyzed the data and visualization processing of characterization technology. Yuanlin Ren and Xiaohui Liu participated in the revision and review of the manuscript.

## Conflicts of interest

The authors listed emphasized that they did not have any potential competing economic interests, and they all approved the accompanying manuscript.

## Supplementary Material
